# SOX21-AS1 is associated with clinical stage and regulates cell proliferation in nephroblastoma

**DOI:** 10.1042/BSR20190602

**Published:** 2019-05-17

**Authors:** Jingxiu Zhang, Tianzhao Hou, Xueliang Qi, Jihong Wang, Xiangguo Sun

**Affiliations:** 1 Department of Pediatrics, Linyi Central Hospital, Linyi, Shandong 276400, China; 2 Department of Surgery, Maternal and Child Health Care Family Planning Service Center of Yishui County, Linyi, Shandong 276400, China

**Keywords:** biomarker, lncRNA, nephroblastoma, SOX21-AS1, Wilms tumor

## Abstract

LncRNA SOX21 antisense RNA 1 (SOX21-AS1) dysregulated in many types of human cancer, and functioned as tumor suppressor or promoter depending on tumor types. However, there was no report about the role of SOX21-AS1 in nephroblastoma. In the present study, we first found that SOX21-AS1 expression was elevated in nephroblastoma tissues and cell lines compared with adjacent normal tissues and normal human embryonic kidney cell line, respectively. Moreover, we observed nephroblastoma patients with large tumor size, advanced National Wilms Tumor Study (NWTS) stage or unfavorable histopathological type, and patients that had higher SOX21-AS1 expression levels than nephroblastoma patients with small tumor size, early NWTS stage or favorable histopathological type. The *in vitro* studies suggested that knockdown of SOX21-AS1 suppressed nephroblastoma cell proliferation and colony formation, and induced cell-cycle arrest through up-regulating p57 expression. In conclusion, our study suggests that SOX21-AS1 functions as oncogenic lncRNA in nephroblastoma, which may provide a novel insight for nephroblastoma carcinogenesis.

## Introduction

Nephroblastoma, also known as Wilms’ tumor, is the most common pediatric kidney cancer accounting for approximately 8% of all malignant tumors in children [1]. The incidence rate of nephroblastoma is approximately 2/100 million [2]. Nephroblastoma is generally discovered under the age of 5 years, and the average age of morbidity is approximately 3 years [3]. Recent decades, the overall survival rate of nephroblast cases has risen to exceeding 90% due to the advance of systemic treatment including surgery, radiotherapy and chemotherapy [4]. In China, the rapid population growth is resulting in a large number of new nephroblastoma cases, which cause serious influence on children’s health [5,6]. Thus, a better understanding of nephroblastoma progression would be a substantial advancement for identifying sensitive molecular biomarkers to predict high risk patients and guide treatment.

Long non-coding RNA (lncRNA) is class of non-coding RNAs with >200 nucleotides in length and no protein coding capacity [7,8]. Recently, lncRNA H19 and LINC00473 have been confirmed to be involved in the development and progression of nephroblastoma [9–13].

LncRNA SOX21 antisense RNA 1 (SOX21-AS1) is located at chromosome 13q32.1 and transcribed into a 2986 nt transcript [14]. In Alzheimer’s disease, knocking down SOX21-AS1 alleviated neuronal oxidative stress and suppressed neuronal apoptosis through up-regulating FZD3/5 and activating Wnt signaling pathway [15]. Moreover, increasing studies showed that SOX21-AS1 functioned as cancer-associated lncRNA in several kinks of human tumor, such as oral cancer [16], lung cancer [17], colorectal cancer [18,19], hepatocellular carcinoma [20] and glioblastoma [21]. However, there was no report about the role of SOX21-AS1 in nephroblastoma. In our early study, five potential associated lncRNAs were screened and measured in nephroblastoma tissues and adjacent normal tissues, and SOX21-AS1 expression level was the highest in nephroblastoma tissues among five lncRNAs. Therefore, the aim of the present study was to explore the clinical value and biological function of SOX21-AS1 in nephroblastoma.

## Materials and methods

### Ethical statement

The present study was approved by the Ethical Review Committee of Linyi Central Hospital and Maternal and Child Health Care Family Planning Service Center of Yishui County. Written informed consent was obtained from all the patients. The present study was carried out in accordance with the World Medical Association Declaration of Helsinki

### Sample collection

Forty pairs of nephroblastoma tissues and corresponding adjacent normal tissues were collected from Linyi Central Hospital or Maternal and Child Health Care Family Planning Service Center of Yishui County. All specimens were confirmed by at least two pathologists, and stored at −80°C refrigerator. All cases have not received preoperative chemotherapy or radiotherapy.

### Cell lines

The nephroblastoma cell lines (WiT49 and WT-CLS1) and normal human embryonic kidney cell line (HEK293) were cultured in Dulbecco’s Modified Eagle Medium (DMEM, Thermo Fisher Scientific, Waltham, MA, U.S.A.) supplemented with 10% FBS (Gibco, Rockville, MD, U.S.A.) at a humidified incubator with 5% CO_2_ and 37°C.

### RNA extraction and quantitative real-time PCR

The total RNAs were extracted from nephroblastoma tissues or cells with TRIzol reagent (Invitrogen, Carlsbad, CA, U.S.A.) according to the manufacturer’s instruction. Then, total RNAs were used for template to reverse-transcribed into cDNAs using PrimeScript RT Master Mix (Takara Biomedical Technology, Beijing, China). The quantitative real-time PCR (qRT-PCR) was conducted with One Step TB Green PrimeScript RT-PCR Kit (Takara Biomedical Technology, Beijing, China) at ABI PRISM 7000 Sequence Detection System (Applied Biosystems, Foster City, CA, U.S.A.) following the manufacturer’s protocols. Gene expression was normalized to GAPDH.

### RNA interference

The SOX21-AS1-specific small interfering RNA (siRNA-SOX21-AS1) and negative control siRNA (siRNA-NC) were purchased from GenePharma Co., Ltd (Shanghai, China). The siRNAs were transfected into nephroblastoma cells through Lipofectamine RNAiMAX transfection reagent (Invitrogen, Carlsbad, CA, U.S.A.) according to the manufacturer’s instruction.

### Cell counting kit-8 (CCK-8) assay

The Cell counting kit-8 (CCK-8) assay was utilized to estimate the effect of SOX21-AS1 on nephroblastoma cell proliferation according to the manufacturer’s instructions. The nephroblastoma cells were transfected with siRNA-SOX21-AS1 for 1, 2, 3 or 4 days, and 10 μl of CCK-8 was added to the culture medium in each well of 96-well plates. After 1 h of incubation at 37°C, the optical density at 450 nm was detected at a microplate monitor.

### Colony formation assay

The transfected nephroblastoma cells (500 cells/well) were plated in six-well plates, and replacing the culture medium every 4 days. After 10 days culture, the colonies were fixed with methanol, and stained with 0.1% Crystal Violet (Beyotime, Shanghai, China). Finally, the specific number of colonies (>50 cells) was manually counted at light microscope.

### Cell cycle assay

After 48-h transfection, the nephroblastoma cells were fixed in ethanol for 1 h and then incubated with RNase A. Afterward, nephroblastoma cells were stained with propidium iodide (Sigma-Aldrich, Louis, MO, U.S.A.) for 30 min at room temperature without light, and were analyzed by using flow cytometer (BD Biosciences, U.S.A.). The percentages of cells in the G0-G1, S, and G2-M phases were counted and compared.

### Western blot

The proteins were extracted from nephroblastoma cells by using RIPA Lysis Buffer (Beyotime, Shanghai, China), and quantified through BCA protein assay kit (Beyotime, Shanghai, China). Equal quantities of protein were isolated by 10% sodium dodecyl sulfate polyacrylamide gel electrophoresis, and then transferred onto polyvinylidene fluoride. Subsequently, membranes were blocked in 5% non-fat milk for 1 h at room temperature, and incubated with anti-p57 or anti-β-actin (Cell Signaling Technology, Danvers, MA, U.S.A.) overnight at 4°C. Next, membranes were incubated with horseradish peroxidase-conjugated secondary antibody for 1 h, and visualized using an enhanced chemiluminescence detection system (Beyotime, Shanghai, China). Finally, the bands were quantified by densitometry using Quantity One software.

### Statistical analysis

All data were presented from at least three independent experiments and analyzed by SPSS 19.0 software (SPSS Inc., Chicago, IL, U.S.A.). The two-tailed Student’s *t*-test was used to estimate the statistical significance between two groups. *P* value <0.05 was considered statistically significant.

## Results

### The expression of SOX21-AS1 is up-regulated in nephroblastoma

**Figure 1:**
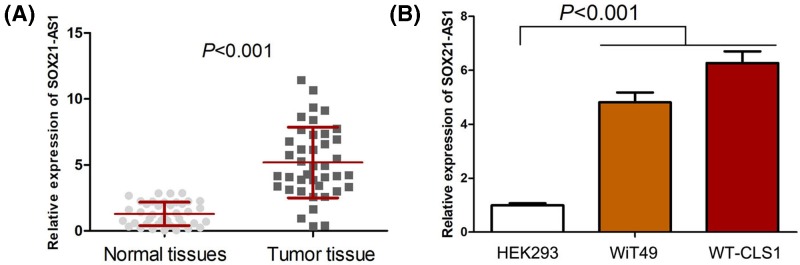
The expression of SOX21-AS1 is up-regulated in nephroblastoma (**A**) SOX21-AS1 expression was remarkably increased in nephroblastoma tissues (*n*=40) compared with corresponding adjacent normal tissues (*n*=40). (**B**) SOX21-AS1 expression in nephroblastoma cell lines was higher than normal human embryonic kidney cell line.

To identify SOX21-AS1 expression status in nephroblastoma, we measured SOX21-AS1 expression in nephroblastoma tissue samples and cell lines through using qRT-PCR. As shown in the Figure 1A, SOX21-AS1 expression was remarkably increased in nephroblastoma tissues compared with corresponding adjacent normal tissues. We also found nephroblastoma cell lines indicated higher SOX21-AS1 expression than normal human embryonic kidney cell line (Figure 1B).

### The association between SOX21-AS1 expression and clinicopathological features in nephroblastoma patients

**Figure 2:**
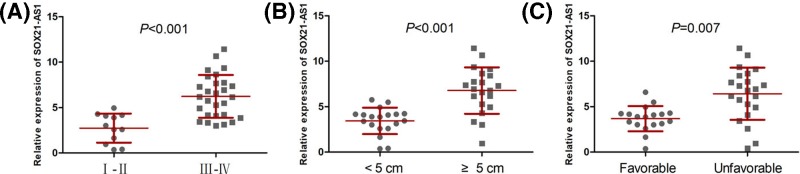
The association between SOX21-AS1 expression and clinicopathological features in nephroblastoma patients (**A**) Nephroblastoma patients with III-IV stage (*n*=28) had higher SOX21-AS1 expression levels than nephroblastoma patients with I-II stage (*n*=12). (**B**) Nephroblastoma patients with ≥5 cm (*n*=21) had higher SOX21-AS1 expression levels than nephroblastoma patients with <5 cm (*n*=19). (**C**) Nephroblastoma patients with unfavorable histopathological type (*n*=22) had higher SOX21-AS1 expression levels than nephroblastoma patients with favorable histopathological type (*n*=18).

In order to know the clinical value of SOX21-AS1 in patients with nephroblastoma, all nephroblastoma patients were classified into different groups based on clinicopathological features including tumor size (<5 cm vs. ≥5 cm), NWTS (National Wilms Tumor Study) stage (I-II vs. III-IV) and histopathological type (favorable vs. unfavorable). As shown in the Figure 2A–C, nephroblastoma patients with ≥5 cm, III-IV or unfavorable had profoundly higher SOX21-AS1 expression levels than nephroblastoma patients with <5 cm, I-II or favorable, respectively.

### Knockdown of SOX21-AS1 suppresses nephroblastoma cell proliferation and colony formation, and induces cell-cycle arrest

**Figure 3:**
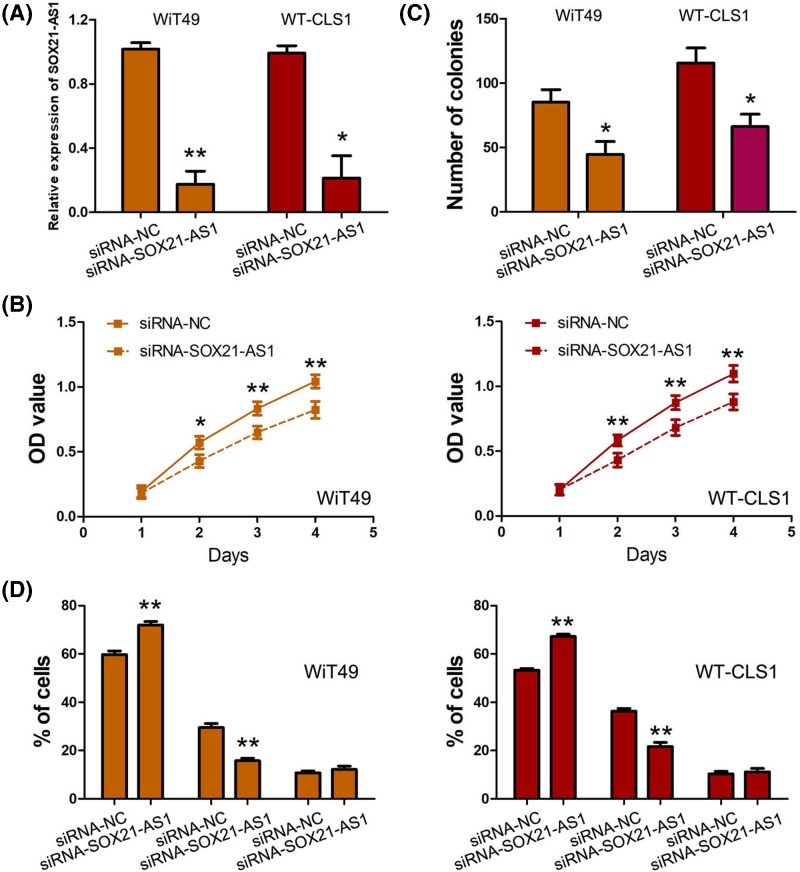
The biological function of SOX21-AS1 in nephroblastoma cells (**A**) The efficient silencing of SOX21-AS1 in nephroblastoma cells was confirmed by using qRT-PCR. (**B**) Knockdown of SOX21-AS1 suppressed nephroblastoma cell proliferation. (**C**) Knockdown of SOX21-AS1 expression inhibited the number of colony formation in nephroblastoma cells. (**D**) Knockdown of SOX21-AS1 expression induced the cycle arrest at G1/G0 phase in nephroblastoma cells. (*, *P*<0.01; **, *P*<0.001).

As shown in the Figure 1B, SOX21-AS1 expression levels were markedly low in nephroblastoma cell lines. Thus, we performed loss-of-function study through siRNA-SOX21-AS1 transfection in nephroblastoma cells. The efficient silencing of SOX21-AS1 in nephroblastoma cells was confirmed by using qRT-PCR (Figure 3A). We conducted CCK-8 assay to assess the effect of SOX21-AS1 expression on nephroblastoma cell proliferation, and found silencing SOX21-AS1 expression obviously impaired cell proliferation ability of nephroblastoma cells (Figure 3B). Moreover, we conducted the colony formation assay to further confirm the role of SOX21-AS1 on nephroblastoma cell growth. Similarly, we observed that silencing SOX21-AS1 expression inhibited the number of colony formation in nephroblastoma cells (Figure 3C). In addition, cycle analysis by flow cytometry suggested that silencing SOX21-AS1 expression induced the cycle arrest at G1/G0 phase in nephroblastoma cells (Figure 3D).

### Knockdown of SOX21-AS1 promotes p57 expression in nephroblastoma cells

**Figure 4:**
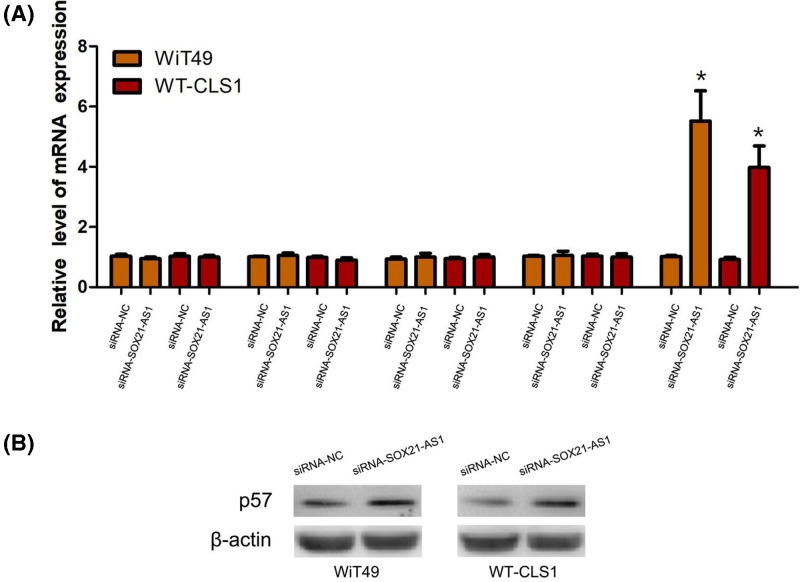
The molecular mechanism of SOX21-AS1 in nephroblastoma cells (**A**) The mRNA levels of p15, p16, p21, p27 and p57 were determined using qRT-PCR following silencing SOX21-AS1 expression in nephroblastoma cells. (**B**) Knockdown of SOX21-AS1 expression increased p57 protein expression in nephroblastoma cells. (*, *P*<0.01).

To investigate the mechanism of SOX21-AS1 on nephroblastoma cell proliferation, the mRNA levels of p15, p16, p21, p27 and p57 were determined using qRT-PCR following silencing SOX21-AS1 expression in nephroblastoma cells. The results of qRT-PCR showed that silencing SOX21-AS1 expression definitely elevated mRNA level of p57, but had no effect on mRNA levels of p15, p16, p21 and p27 (Figure 4A). Furthermore, we performed Western blot to confirm the influence of SOX21-AS1 on the p57 protein expression, and found that silencing SOX21-AS1 expression remarkably increased p57 protein expression in nephroblastoma cells (Figure 4B).

## Discussion

SOX21-AS1 has been showed to be dysregulated in many types of human cancer. Originally, Yang et al. [16] comprehensively analyzed transcriptome profiles from two pairs of oral squamous cell carcinoma and corresponding adjacent normal tissues, and identified SOX21-AS1 for the following study due to a CpG-rich region upstream of SOX21-AS1. Then, SOX21-AS1 expression levels were found to be down-regulated in oral squamous cell carcinoma tissue samples compared with matching normal tissue samples [16]. Furthermore, the clinical correlation analysis showed low SOX21-AS1 expression was correlated with advanced clinical stage and large tumor size, and univariate and multivariate Cox’s regression analysis suggested low SOX21-AS1 expression acted as an independent unfavorable prognostic factor for disease-specific survival in patients with oral squamous cell carcinoma [16]. Afterward, Paul et al. [21] also performed lncRNA transcript profiling from 19 glioblastoma samples and 9 control brain samples, but found there was no significant difference of SOX21-AS1 expression between glioblastoma samples and control brain samples. Furthermore, they performed survival analysis from the TCGA glioblastoma RNA-Seq data, and identified SOX21-AS1 as a good prognostic predictor for overall survival in glioblastoma patients [21]. On the contrary, recent more evidence suggested SOX21-AS1 expression was increased in human cancers. Lu et al. [17] performed and analyzed TCGA lung cancer RNA-Seq data, and found that SOX21-AS1 expression was increased approximately 5-fold in lung cancer tissues compared with normal lung tissues. Then, they confirmed high SOX21-AS1 expression status in lung cancer tissues and cell lines through qRT-PCR, and observed patients with high SOX21-AS1 expression tended to have large tumor size or advanced TNM stage [17]. Besides, Kaplan–Meier survival analysis suggested higher SOX21-AS1 expression correlated with worse overall survival, and univariate and multivariate analyses indicated that high SOX21-AS1 expression was an independent biomarker for unfavorable overall survival in lung cancer patients [17]. Similarly, Wei et al. [20] found SOX21-AS1 expression was increased in hepatocellular carcinoma tissues and cell lines respectively compared with adjacent normal liver tissues and normal liver epithelial cell line, and SOX21-AS1 overexpression was associated with large tumor size, high Edmonson grade, vascular invasion and cirrhosis in patients with hepatocellular carcinoma. The survival analyses revealed that cases with high SOX21-AS1 expression had short survival time compared with cases with low SOX21-AS1 expression, and high SOX21-AS1 expression served as an independent unfavorable predictor for overall survival in patients with hepatocellular carcinoma [20]. In colon cancer, Wang et al. [18] comprehensively analyzed TCGA colon cancer RNA-Seq data, and found that SOX21-AS1 expression was obviously up-regulated in colon cancer tissues. In addition, Wei et al. [19] performed qRT-PCR to confirm high levels of lncRNA SOX21-AS1 in colorectal cancer tissue samples and cell lines. Meanwhile, they observed aberrant high SOX21-AS1 expression predicted unfavorable clinical outcome in colorectal cancer patients [19]. The expression pattern and clinical significance of SOX21-AS1 were still unknown in nephroblastoma. In our study, we first found that SOX21-AS1 expression was elevated in nephroblastoma tissues and cell lines compared with adjacent normal tissues and normal human embryonic kidney cell line, respectively. Moreover, we observed nephroblastoma patients with large tumor size, advanced NWTS stage or unfavorable histopathological type had higher SOX21-AS1 expression levels than nephroblastoma patients with small tumor size, early NWTS stage or favorable histopathological type, respectively.

SOX21-AS1 functioned as tumor suppressor or promoter depending on tumor types. In oral cancer cells, Yang et al. [16] indicated that SOX21-AS1 overexpression markedly inhibited cell proliferation and invasion. However, Wei et al. [20] found that down-regulation of SOX21-AS1 expression inhibited proliferation and invasion, arrested cell cycle process, and accelerated cell apoptosis through increasing p21 expression in hepatocellular carcinoma. In colorectal cancer cells, Wei et al. [19] that showed SOX21-AS1 sponged miR-145 to enhance tumor cell proliferation and invasion *in vivo*, and tumor growth *in vitro* through up-regulating myosin VI. Moreover, Lu et al. [17] found that inhibition of SOX21-AS1 expression repressed lung cancer cell proliferation and cell cycle progression through up-regulating p57 expression. Similarly, we also found knockdown of SOX21-AS1 suppressed nephroblastoma cell proliferation and colony formation, and induced cell-cycle arrest through elevating p57 expression.

In conclusion, SOX21-AS1 expression is increased in nephroblastoma tissues and cells, and has relationships with tumor size, NWTS stage and histopathological type in nephroblastoma cases. Down-regulation of SOX21-AS1 expression mediates p57 to inhibit nephroblastoma cell proliferation, colony formation and cell-cycle progression.

## Abbreviations

CCK-8: cell counting kit-8
lncRNA: long non-coding RNA
NWTS: National Wilms Tumor Study
qRT-PCR: quantitative real-time PCR
SOX21-AS1: SOX21 antisense RNA 1
